# A Salient Object Detection Method Based on Boundary Enhancement

**DOI:** 10.3390/s23167077

**Published:** 2023-08-10

**Authors:** Falin Wen, Qinghui Wang, Ruirui Zou, Ying Wang, Fenglin Liu, Yang Chen, Linghao Yu, Shaoyi Du, Chengzhi Yuan

**Affiliations:** 1 School of Physics and Mechanical and Electrical Engineering, Longyan University, Longyan 364012, China; wenfalin2008@163.com (F.W.); wqh0597@126.com (Q.W.); zray_zou@126.com (R.Z.); lyxyy_w@126.com (Y.W.); liufenglin45@126.com (F.L.); chenyang4117@163.com (Y.C.); 2 School of Software Engineering, Xi’an Jiaotong University, Xi’an 710049, China; 13807274715@163.com; 3 Institute of Artificial Intelligence and Robotics, Xi’an Jiaotong University, Xi’an 710049, China; dushaoyi@xjtu.edu.cn; 4 Department of Mechanical, Industrial and Systems Engineering, University of Rhode Island, Kingston, RI 02881, USA

**Keywords:** salient object detection, multi-level features, multi-scale information, boundary enhancement

## Abstract

Visual saliency refers to the human’s ability to quickly focus on important parts of their visual field, which is a crucial aspect of image processing, particularly in fields like medical imaging and robotics. Understanding and simulating this mechanism is crucial for solving complex visual problems. In this paper, we propose a salient object detection method based on boundary enhancement, which is applicable to both 2D and 3D sensors data. To address the problem of large-scale variation of salient objects, our method introduces a multi-level feature aggregation module that enhances the expressive ability of fixed-resolution features by utilizing adjacent features to complement each other. Additionally, we propose a multi-scale information extraction module to capture local contextual information at different scales for back-propagated level-by-level features, which allows for better measurement of the composition of the feature map after back-fusion. To tackle the low confidence issue of boundary pixels, we also introduce a boundary extraction module to extract the boundary information of salient regions. This information is then fused with salient target information to further refine the saliency prediction results. During the training process, our method uses a mixed loss function to constrain the model training from two levels: pixels and images. The experimental results demonstrate that our salient target detection method based on boundary enhancement shows good detection effects on targets of different scales, multi-targets, linear targets, and targets in complex scenes. We compare our method with the best method in four conventional datasets and achieve an average improvement of 6.2% on the mean absolute error (MAE) indicators. Overall, our approach shows promise for improving the accuracy and efficiency of salient object detection in a variety of settings, including those involving 2D/3D semantic analysis and reconstruction/inpainting of image/video/point cloud data.

## 1. Background

In recent years, deep convolutional neural networks (CNNs) have demonstrated exceptional performance in various visual tasks and are highly valuable for applications. However, they often perform poorly when evaluated on datasets with different distributions from the training set and are susceptible to overfitting. To enhance the model’s generalization, numerous data augmentation methods and regularization strategies have been proposed.

Salient target detection involves detecting and segmenting the most noticeable object area in an image, making it a two-stage visual task. Although most methods perform both stages simultaneously, this paper takes a deep learning approach that captures different levels of features for salient target detection. By training on a dataset and utilizing the image’s salient information to generate diverse data at the feature level, our proposed model avoids generating interference data while maintaining the key information of the image. This approach improves the model’s performance on the test set.

Salient object detection (SOD) is a fundamental problem in computer vision, which aims to automatically identify and localize the most visually conspicuous regions in an image or a scene. The demand for SOD has been steadily growing due to its wide-ranging application in various fields.

In the field of medical imaging, SOD plays a critical role in assisting medical professionals in diagnosing diseases and abnormalities. By accurately detecting salient regions in medical images, such as MRI or CT scans, SOD can aid in identifying important structures, lesions, or anomalies, improving medical diagnosis and treatment planning. In robotics and autonomous systems, visual attention mechanisms inspired by human saliency are of paramount importance. For robots to efficiently navigate and interact with their environment, they need to focus on relevant objects or regions. SOD helps robots prioritize salient visual information, enhancing their decision-making and perception capabilities. Furthermore, SOD finds applications in video analysis and surveillance, where the detection of salient objects in a sequence of frames can aid in tracking moving objects or identifying potential threats. Additionally, in fields like human–computer interaction and augmented reality, SOD contributes to creating more immersive and intuitive user experiences by highlighting relevant elements of interest. The ability to efficiently detect salient objects is also valuable in image and video editing tasks, such as object removal, background inpainting, and image retouching. By accurately identifying salient regions, these editing tasks can be performed more effectively and with higher visual quality.

Overall, the significance of SOD in image processing lies in its ability to mimic human visual attention, enabling efficient and effective analysis of visual information. By identifying salient objects and regions, SOD improves the performance and accuracy of various image-related applications, including medical diagnosis, robotics, human–computer interaction, and content-based retrieval. Its integration into these fields can lead to breakthroughs in technology, making systems more intuitive, responsive, and capable of handling complex real-world scenarios. Given the diverse applications of SOD, there is a growing demand for robust and efficient algorithms that can handle different types of images, scenes, and visual challenges. The proposed method based on boundary enhancement addresses some of the limitations of existing techniques and offers improved detection performance, making it a valuable contribution to the field.

## 2. Problem Description

Scale variation constitutes one of the key challenges of the SOD task, which is hard for CNNs to handle, owing to the constraint of the downsampling operation. Different layers of features only possess the ability to handle specific scales, and the level of target information contained in features with different resolutions varies. In the top-down path, the features of every layer are produced horizontally, and the approach of upsampling to a unified resolution and then merging only utilizes a separate resolution feature in each layer, which falls short of coping with problems of various scales. To tackle these issues, the Amulet network employs shrinking and expanding methods to change feature sizes and fuses all levels of features at each level in the transmission layer to make effective use of multi-scale information [1]. However, this fusion method tends to generate redundant information and noise interference. Additionally, atrous spatial pyramid pooling (ASPP) [2] and pyramid pooling module (PPM) [3] are two common options for multi-scale information extraction, typically fixed at the last layer of network feature extraction. These methods also struggle to effectively handle small-scale objects as deep features, especially top-level features, which contain less information regarding such objects.

The feature extraction process in pixel-level saliency often results in the loss of detailed information and unsatisfactory boundary areas for salient targets. To address this issue, some methods recursively refine high-level features using low-level local information [4,5]. The method proposed by Zhao et al. [6] provided direct supervision on salient object boundary detection, taking into account both salient boundary information and salient objects. Other methods [7,8] used superpixels for preprocessing to extract boundaries before saliency detection or adopt conditional random field (CRF) for postprocessing on saliency prediction maps to preserve object boundaries. However, these methods require additional processing and have relatively low efficiency. Cross-entropy loss is a commonly used loss function but treats each pixel equally without considering the boundary pixels, leading to blurred boundaries. The method proposed by Qin et al. [9] addressed this issue by modifying the loss function to emphasize the pixels in the border area.

This study proposes a salient object detection method based on boundary enhancement to address the challenges in salient object detection. The method builds on the Feature Pyramid Network (FPN) [10] using a multi-level feature aggregation module to aggregate features from adjacent layers and enhance the expressive power of features at different resolutions. A multi-scale information extraction module is then applied to further improve the expressive ability of features. The module is implemented in each layer of the decoder and can be easily adapted to other network models. In addition, the method explicitly models the boundary of salient objects and fuses salient boundary information with salient object information to supplement the salient object information. The method utilizes a set of mixed losses for supervised training of the saliency detection task, further enhancing the detection effect and highlighting the salient regions uniformly. The proposed method is evaluated on four public saliency datasets and outperforms many existing mainstream methods on multiple indicators.

Certain techniques, such as multi-level feature aggregation and multi-scale information extraction [11,12,13], have been explored in prior research on SOD. While the proposed method shares common modules with these earlier publications, the main contributions of our method lie in the innovative combination and adaptation of these techniques to address specific challenges in SOD. The primary contributions of our work can be summarized as follows. (1) Boundary Enhancement Technique: Our method introduces a novel boundary extraction module, which enhances the detection accuracy of salient objects by effectively capturing the boundary information of the regions of interest. This innovative technique improves the precision and robustness of the saliency prediction results. (2) Multi-level Feature Aggregation: While multi-level feature aggregation has been used in previous works, our method introduces a specific multi-level feature aggregation module that addresses the challenge of large-scale variation of salient objects. It enhances the expressive ability of fixed-resolution features by effectively utilizing adjacent features to complement each other, leading to improved detection results. (3) Multi-scale Information Extraction: Although multi-scale information extraction has been explored in the literature, our method proposes a novel approach to capture local contextual information at different scales for back-propagated level-by-level features. This enables better measurement of the composition of the feature map after back-fusion, resulting in more accurate saliency predictions. (4) Boundary and Salient Target Fusion: The proposed method introduces a boundary and salient target fusion technique that refines the saliency prediction results by addressing the low confidence issue of boundary pixels. This fusion enhances the overall accuracy of the salient object detection.

## 3. Salient Object Detection Based on Boundary Enhancement

**Figure 1:**
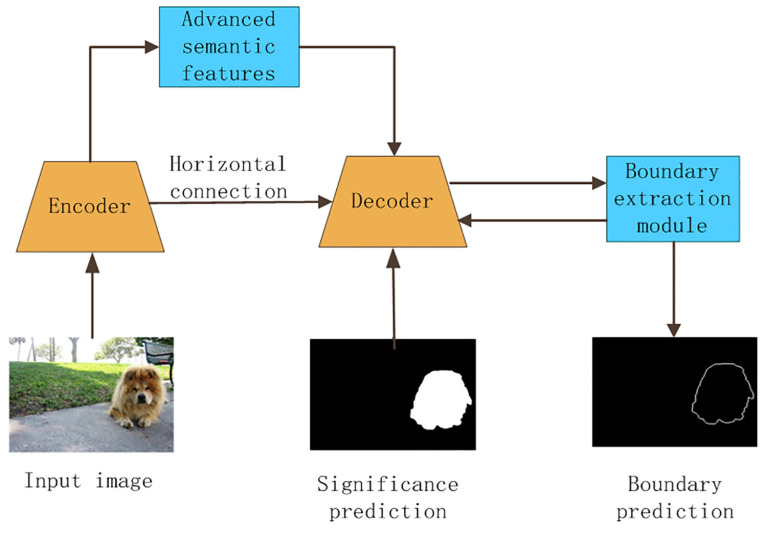
The overall framework of the salient target detection method based on boundary enhancement.

The use of CNNs can lead to loss of necessary information during the acquisition of advanced semantic features through continuous subsampling. This will result in inaccurate and inconsistent segmentation of salient objects of different scales. To address these issues, this paper proposes a network model based on the FPN structure, which combines bottom-up and top-down approaches to effectively integrate features from different levels. The proposed model also incorporates advanced semantic features for localizing salient objects and low-level features for refining deep-layer features. Additionally, the model explicitly models the boundaries of salient objects and fuses the extracted boundary information with the salient object information to improve the overall detection accuracy. A set of mixed losses is used for supervised training to further enhance the detection effect and uniformly highlight the salient regions. Figure 1 shows the overall framework of the proposed salient target detection model based on boundary enhancement.

The feature extraction process follows these steps: First, an input image is processed by the encoder to extract multi-level features. Then, the decoder and horizontal features are progressively fused from top to bottom, and the resulting fused features are fed into the boundary extraction module to extract the boundary information. This information is then sent back to the decoder, where it is merged with the salient target information, resulting in a pixel-level salient target prediction map.

### 3.1. Feature Extraction Network

**Figure 2:**
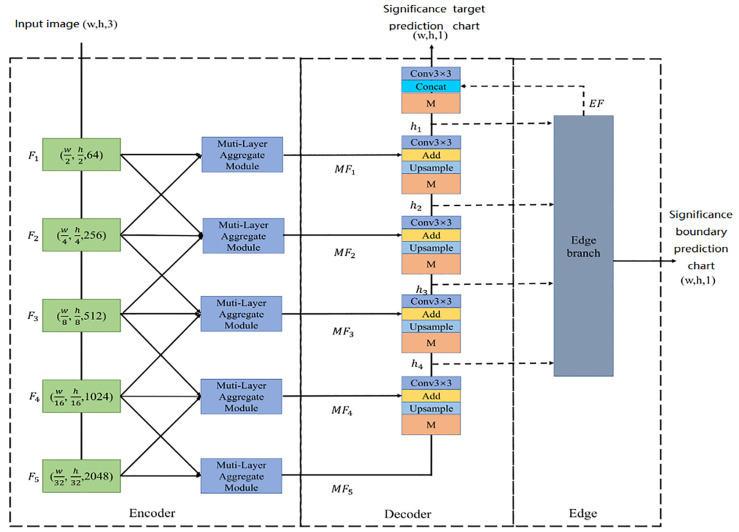
Detailed network structure diagram, taking ResNet-50 as an example.

As shown in Figure 2, the feature extraction network consists of two stages: the first stage is the encoder feature extraction stage, which uses the feature extraction network to extract features and abstract information at different resolutions, and aggregates adjacent features once using a multi-level feature aggregation module. The second stage is the decoder reverse fusion stage, which uses a multi-scale information extraction module in the reverse fusion process, extracts higher-level semantic information and multi-scale information, and fuses it with the multi-level aggregation features of the previous level after upsampling. In addition, in the decoder stage, the boundary extraction module is used to extract the boundary of salient targets on features containing multi-scale information. The boundary information is used to supplement the salient target information, thereby obtaining a more accurate salient target prediction map. Figure 1 illustrates the overall framework of our method, showcasing the interactions between the encoder, decoder, and boundary extraction modules. Additionally, the edge section, as depicted in Figure 2, is connected to the boundary extraction module, contributing to the method’s boundary enhancement capability. Moreover, the multi-scale information extraction module, a critical component of our approach, captures local contextual information at different scales for improved saliency predictions.

- Encoder

During the common feature extraction process in the encoder stage, VGG-16 and ResNet-50 are used as backbones in our model. We directly remove the last pooling and fully connected layers of the VGG-16 and ResNet-50 models. For example, using ResNet-50, the input image is processed through the backbone network to extract five sets of convolutional layers, resulting in five feature maps F1-F5 with different resolutions and channel numbers. If VGG-16 is used as the backbone, the sizes of the five feature maps obtained are 1/1, 1/2, 1/4, 1/8, and 1/16 of the original image, respectively. The feature maps vary in size from 1/2 to 1/32, with the number of channels being 64, 256, 512, 1024, and 2048, respectively, from F1 to F5. From F1 to F5, the level of detail decreases while the level of semantic information increases. As shown in the encoder stage on the left side of Figure 2, the input image is processed through the backbone network to extract five features with different resolutions and channel numbers.

To enhance the extraction of effective information from fixed-resolution features, we modified the encoder section by adding a new multi-level feature aggregation module to the transmission layer. In the encoder stage, depicted in the left section of Figure 2, every level of features is input into the feature aggregation module, along with the higher and lower-level features, to enhance the expressive ability of various resolution features. Subsequently, we obtained five multi-level aggregation features, denoted as MF1-MF5, which are transmitted to the decoder as the corresponding horizontal output at each level.

- Decoder

The second stage is the reverse fusion phase, which aims to gradually fuse features extracted by the encoder with varying resolutions and channel numbers. This approach maximizes the use of information from different scale features and integrates them to perform upsampling, eventually restoring the salient target prediction map to the original image size. Instead of directly upsampling features of different sizes and restoring them to the size of the original image, then splicing and merging, the reverse fusion process is conducted step by step from top to bottom. Each operation only requires merging two features with similar resolutions, thereby reducing the introduction of noise and producing more accurate results.

The decoder stage in Figure 2 illustrates the reverse level-by-level fusion process using ResNet-50 as an example. The process starts from the highest-level multi-level feature MF5 and proceeds sequentially downwards. The feature is first input into the multi-scale information extraction module to extract its multi-scale information, followed by upsampling it to twice its original size. The channel dimension is then reduced using 1×1 convolution to ensure that the size and channel dimension of the multi-scale feature match those of the upper-level horizontal features. This approach helps to reduce the parameters and calculations. The feature is then added directly to the elements of the low-level horizontal features for fusion. Finally, a 3×3 convolution is applied to further abstract the features and obtain the fusion feature map, which is then input to the next level.

After performing the reverse fusion stage, the resulting feature maps $h_{4}$, $h_{3}$, $h_{2}$, and $h_{1}$ contain rich multi-scale information. These feature maps are then passed through the boundary extraction module to obtain the feature EF, which contains the boundary information of the salient object. The feature map $h_{1}$ is also passed through the multi-scale extraction module to extract multi-scale information, which is then concatenated with the boundary feature EF along the channel dimension. After reducing the channel dimension with a 1×1 convolution, the feature map is upsampled to restore the original image size and passed through a Sigmoid function to obtain the final saliency target prediction image. The reverse step-by-step fusion process is shown in Equation (1).

(1) $$h_{i}=\left\{\begin{array}{ccc} MF_{i}, & \; & i=5\\ Conv_{3\times 3}\left(Conv_{1\times 1}\left(Up\left(M\left(h_{i+1},2\right)\right)\right)+MF_{i}\right), & \; & 1\leq i\leq 4 \end{array}\right.$$

where $h_{i+1}$ represents the feature map generated at each level in the backward fusion process; $MF_{i}$ represents the multi-level aggregated features at different scales in the horizontal connection; Up(.,2) represents an upsampling operation, which doubles the size of the input feature map each time; $Conv_{3\times 3}\left(\cdot \right)$ represents the 3×3 convolution operation; $Conv_{1\times 1}\left(\cdot \right)$ represents the 1×1 convolution operation; and M(·) represents the multi-scale information extraction module.

In the proposed SOD method, several potential pre-trained backbone models were considered for feature extraction. Some of the commonly used backbone architectures include VGG-16, ResNet-50, EfficientNet, and Inception, among others. VGG-16 and ResNet-50 were selected as the backbone models for the study for the following reasons: (1) VGG-16 is a widely used deep convolutional neural network architecture known for its simplicity and effectiveness. It consists of 16 layers, primarily composed of small 3 × 3 convolutional filters, making it easy to implement and interpret. Despite its straightforward structure, VGG-16 has shown excellent performance in various computer vision tasks, making it a reliable choice as a backbone for salient object detection. (2) ResNet-50 is a deeper architecture that introduces the concept of residual connections to address the vanishing gradient problem in deep neural networks. With its skip connections, ResNet-50 enables the training of very deep networks, allowing for more complex feature representations. This architecture has achieved remarkable results in image recognition tasks and has become a standard choice for various computer vision applications.

The impact of using another backbone model, such as EfficientNet or Inception, can be substantial and can influence the performance of the salient object detection method. The following are some potential impacts. (1) Computational Efficiency: EfficientNet is specifically designed to achieve better efficiency by scaling the model’s depth, width, and resolution in a balanced manner. Using EfficientNet as a backbone could lead to faster inference times and lower memory requirements compared to VGG-16 and ResNet-50. (2) Feature Representation: Different backbone models capture different levels of feature representations. Inception, for example, incorporates various convolutional filter sizes to capture multiple scales of information. Using a different backbone could alter the feature extraction capabilities, potentially affecting the detection accuracy and robustness. (3) Generalization: The choice of backbone model may impact the generalization of the salient object detection method to unseen data. Some backbones may generalize better to diverse datasets, while others may perform better on specific datasets based on the nature of the learned features. (4) Transfer Learning: The choice of the backbone model can affect the ease of transfer learning. Pre-training on large-scale datasets and fine-tuning on specific SOD datasets might require different strategies, depending on the backbone’s architecture.

Overall, the choice of the backbone model depends on a trade-off between computational efficiency, feature representation, and the specific requirements of the application. While VGG-16 and ResNet-50 have shown good performance in our study, using other backbones like EfficientNet or Inception may lead to different trade-offs and performance outcomes. It is essential to experiment with various backbone architectures to find the best fit for a particular salient object detection task or dataset.

### 3.2. Multi-Level Feature Aggregation Module

In the process of feature extraction, convolution and pooling operations are used to abstract image features at multiple levels. However, different levels of features have varying degrees of abstraction, and integrating feature information from other levels can enhance their expressive ability. For instance, shallow features can integrate information from high-level features, which can help suppress noise and enhance detailed information. On the other hand, high-level features can integrate feature information from shallow features, which can further enhance semantic information. Some features can also integrate information from both shallow and high-level features. To improve the expressive ability of fixed-resolution features by aggregating multi-level features and further enhance the model’s performance, this paper proposes a multi-level feature aggregation module.

**Figure 3:**
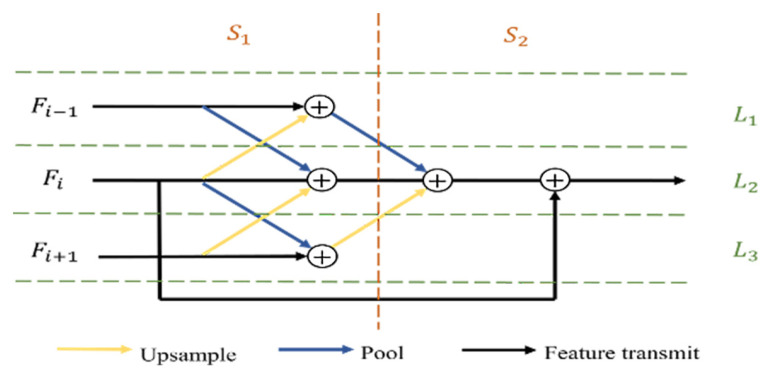
Structure diagram of multi-level feature aggregation module.

The proposed multi-level feature aggregation module, illustrated in Figure 3, consists of two stages: complementarity and aggregation. The resolution of the input features gradually decreases from $F_{i-1}$ to $F_{i+1}$, while the number of channels gradually increases. In the complementary stage ($S_{1}$), the three input features undergo 1×1 convolution to ensure consistency in the number of channels, which reduces computational costs and facilitates subsequent element fusion. Next, feature $F_{i}$ is pooled and upsampled to supplement the information of $F_{i-1}$ to $F_{i+1}$, respectively. Similarly, $F_{i-1}$ to $F_{i+1}$ are pooled and upsampled to complement $F_{i}$. These pooling and upsampling operations are performed so that the complementary features have the same resolution. The preprocessing process is shown in Equation (2), and the feature complementation process is calculated using Equations (3)–(5).

(2) $$F_{j}^{{}^{\prime }}=ReLU\left(Conv\left(F_{j}\right)\right),\;\;j=i-1,i,i+1$$

(3) $$F_{i(S_{1})}^{{}^{\prime }}=F_{i}^{{}^{\prime }}+AvgPool\left(F_{i-1}^{{}^{\prime }}\right)+Up\left(F_{i+1}^{{}^{\prime }}\right)$$

(4) $$F_{i-1(S_{1})}^{{}^{\prime }}=F_{i-1}^{{}^{\prime }}+Up\left(F_{i}^{{}^{\prime }}\right)$$

(5) $$F_{i+1(S_{1})}^{{}^{\prime }}=F_{i+1}^{{}^{\prime }}+AvgPool\left(F_{i}^{{}^{\prime }}\right)$$

where $F_{j}^{{}^{\prime }}$ represents the jth level feature after reducing the channel dimension; $F_{i(S_{1})}^{{}^{\prime }}$ represents the ith level feature in the complementary stage $S_{1}$ after supplementation; Conv(·) represents convolution responsible for changing the channel dimension; ReLU represents the Rectified Linear Unit non-linear activation function; Up(·) represents the upsampling operation; and AvgPool(·) represents the average pooling operation. It is worth noting that the top-level feature $F_{5}$ and the bottom-level feature $F_{1}$ have only one neighbor, so in the complementary stage, they have only two channels, $L_{2}+L_{3}$ and $L_{1}+L_{2}$, respectively.

The feature aggregation stage, $S_{2}$, aggregates complementary features from different channels to obtain horizontal features that contain multi-level information and provide them to the decoder. The calculation process is illustrated in Equation (6).

(6) $$MF_{i}=F_{i}^{{}^{\prime }}+AvgPool\left(F_{i-1(S_{1})}^{{}^{\prime }}\right)+Up\left(F_{i+1(S_{1})}^{{}^{\prime }}\right)+F_{i(S_{1})}^{{}^{\prime }}$$

On the other hand, the features are fused element-wise in both the complementary and aggregation stages. $MF_{1}$ and $MF_{5}$ aggregate the features of only one neighbor. Moreover, all element fusion operations in the aforementioned stages are followed by a set of 3×3 convolutions, which includes a combination of regularization and non-linear variation ReLU to further abstract features.

### 3.3. Multi-Scale Information Extraction Module

Convolutional networks with frequent downsampling operations suffer from a loss of target information, which accumulates with the feature layer. Additionally, the amount of information contained in each level of feature layers varies for targets of different scales, and each convolutional layer can only capture information with a fixed receptive field size. Small objects have scant information in the top-level features, and commonly used top-level multi-scale extraction methods prove insufficient for handling this situation. Meanwhile, the size of salient objects in visual scenes varies significantly, complicating the salient object detection task. Effectively extracting information from data with changing target scales becomes a crucial issue. Therefore, network design must consider how to extract and aggregate multi-scale information more effectively to tackle the challenge of large target scale changes in visual scenes.

**Figure 4:**
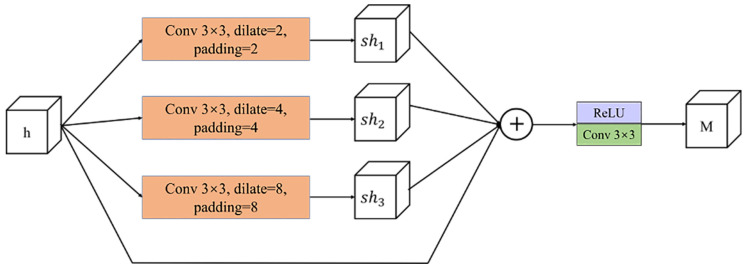
Multi-scale information extraction module.

Based on the aforementioned issues, this section proposes a set of multi-scale information extraction modules inspired by the ASPP idea [2]. These modules are capable of extracting multi-scale information from specific-level features and addressing the problem of target scale changes more effectively. As depicted in Figure 4, each module comprises four branches, out of which three correspond to convolutional layers with distinct hole ratios. These layers can extract features with different receptive fields, containing information of various scales, which are then fused further to obtain the final output featuring multi-scale information.

Specifically, given an input feature h, during the forward process, the feature is first extracted by dilated convolutions with dilation rates of 2, 4, and 8 to obtain features $sh_{1}$, $sh_{2}$, and $sh_{3}$, respectively, which contain information at different scales. The calculation Equation (7) is expressed as follows:

(7) $$sh_{i}=AstrousConv_{3\times 3}\left(h,padding=s,dilate=s\right)\;\;\;s=\left\{\begin{array}{ccc} 2, & \; & i=1\\ 4, & \; & i=2\\ 8, & \; & i=3 \end{array}\right.$$

Here, $AstrousConv_{3\times 3}\left(\cdot \right)$ represents a 3×3 dilated convolution kernel, dilate represents the sampling rate of the dilated convolution, and in order to maintain the same feature resolution, padding is consistent with the dilate.

The second step involves fusing the original features with the extracted multi-scale information using residual operations to preserve the original feature information. The resulting features are further aggregated using a convolution operation and an activation function to improve their non-linear capabilities, and a feature *M* is obtained that contains multi-scale information. Equation (8) expresses the calculation process.

(8) $$M=ReLU(Conv_{3\times 3}\left(\sum_{i=1}^{3}sh_{i}+h\right))$$

where $Conv_{3\times 3}$ indicates a 3×3 convolution operation and $\sum_{i=1}^{3}sh_{i}+h$ is the fusion of element-wise addition of feature layers.

The multi-scale extraction module presented in this paper offers three significant advantages. First, it can capture contextual information of diverse scales in spatial features. Second, it can expand the receptive field without increasing the number of parameters. Lastly, the module has a straightforward structure and can adjust hole rate parameters to suit various datasets. It is also effortless to integrate this module with existing network architectures.

### 3.4. Boundary Extraction Module

**Figure 5:**
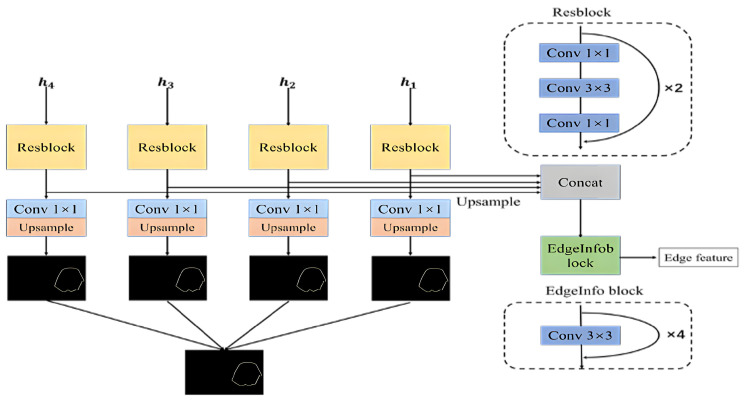
Boundary extraction module.

In this paper, we propose a method for extracting the boundaries of salient objects by constructing an additional branch for boundary detection. This branch runs parallel to the top-down path of the network and takes the features containing multi-scale information generated in the reverse stepwise fusion process as input for the boundary extraction module. By using this approach, we are able to predict the boundaries of the saliency targets. The detailed structure of the proposed approach is illustrated in Figure 5.

Four features, $h_{1},h_{2},h_{3}$, and $h_{4}$, generated in the decoder are utilized as input. They first pass through a ResBlock module for information conversion and channel dimension reduction. Different levels of edge features, $eh_{1},eh_{2},eh_{3}$, and $eh_{4}$, are obtained by 1×1 convolution to change the number of channels to 1, and then upsampled to obtain the saliency boundary prediction map, $e_{1},e_{2},e_{3}$, and $e_{4}$. The boundary of each level is used for network training and forecasting supervision. The ResBlock module is a combination of two residual blocks that have the functions of converting information and reducing channels, as shown in the upper right corner of Figure 5. The calculation process of multi-level boundary extraction is shown in Equations (9) and (10).

(9) $$eh_{i}=ResBlock\left(h_{i}\right)$$

(10) $$e_{i}=Sigmoid\left(Up\left(Conv_{1\times 1}\left(eh_{i}\right),n\right)\right)$$

In these equation, $h_{i}$ represents the feature map generated at each level during the reverse fusion process; $eh_{i}$ represents the saliency boundary feature at each level; $e_{i}$ represents the saliency boundary prediction at each level; Up(·,n) represents the upsampling operation, which upsamples by a factor of *n*; Conv1×1(·) represents the 1×1 convolution operation; ResBlock represents the information transformation module; and Sigmoid(·) represents the activation function.

In order to obtain more precise saliency boundary predictions, we fuse the saliency boundary predictions of different levels, namely, $e_{1},e_{2},e_{3}$, and $e_{4}$. The fused output also needs to be supervised and trained. The calculation formula for this process is shown in Equation (11).

(11) $$e_{fuse}=Conv_{1\times 1}\left(Concat\left(e_{1},e_{2},e_{3},e_{4}\right)\right)$$

where Concat(·) indicates splicing features along the channel; $Conv_{1\times 1}\left(\cdot \right)$ represents the 1×1 convolutional operation, reducing the channel dimension to 1; and $e_{fuse}$ represents a saliency boundary prediction map that fuses multiple levels.

In order to transfer the extracted boundary information of salient targets to the salient target prediction branch to compensate for the missing details, this paper will first upsample the extracted multi-level boundary features, $eh_{1},eh_{2},eh_{3}$, and $eh_{4}$, and then concatenate them along the channel before inputting them into the EdgeInfo module for further fusion to obtain features containing rich boundary information. The structure of EdgeInfo is shown in the lower right corner of Figure 5, which includes four convolutional layers and has the function of further fusing features and changing the number of channels. The calculation process of multi-level boundary feature fusion is shown in Equation (12).

(12) $$EF=EdgeInfo(Concat\left(Up\left(eh_{1}\right),Up\left(eh_{2}\right),Up\left(eh_{3}\right),Up\left(eh_{4}\right)\right))$$

where EdgeInfo(·) represents the boundary feature aggregation module and EF represents the salient boundary features that aggregate multi-level information and can be used to fuse with the salient object features in the next step.

### 3.5. Loss Function

The proposed method uses two categories of loss functions during training to address different tasks: salient boundary detection and salient target detection. Specifically, the salient target detection loss is a mixture of two loss functions. The total loss function used for training is shown in Equation (13):

(13) $$Loss=L_{sod}+\lambda_{1}L_{edge}$$

where $\lambda_{1}$ is a hyperparameter used to balance the losses of the two tasks, and its value is set to 1 in the experiments.

The salient target detection loss function is a combination of two types of loss functions with different focuses. It includes binary cross-entropy loss for individual pixels and consistency-enhancing loss for foreground regions. Its calculation, Equation (14), is expressed as follows:

(14) $$L_{sod}=L_{bce}+L_{cel}$$

(1) Binary Cross Entropy Loss (BCE)

The detection of salient objects commonly utilizes BCE loss as its primary loss function. As a pixel-level loss function, BCE loss does not consider pixel connectivity and therefore cannot differentiate foreground and background pixels. This loss function does not prioritize the pixels in the boundary area or account for the integrity of the target during the training process. Instead, convergence is achieved across all pixels using the following calculation, Equation (15).

(15) $$L_{bce}=\sum_{p\subseteq P,g\subseteq G}^{}-\left[g\log p+\left(1-g\right)\log\left(1-p\right)\right]$$

where *P* represents the predicted saliency map, *p* represents a pixel in *P*, *G* represents the ground truth map, *g* represents a pixel in *G*, and log(·) represents the logarithmic operation at the pixel level.

Consistency Enhanced Loss (CEL): Referencing Intersection over Union (IoU) loss, this loss function uses image-level consistency loss, which can make the loss function more focused on the foreground and less susceptible to scale changes. The calculation Equation (16) and its corresponding gradient calculation Equation (17) of this loss function are expressed as follows:

(16) $$L_{cel}=\frac{\sum_{}^{}\left(p-pg\right)+\sum_{}^{}\left(g-pg\right)}{\sum_{}^{}p+\sum_{}^{}g}$$

(17) $$\frac{\partial L_{cel}}{\partial p}=\frac{1-2g}{\sum_{}^{}\left(p+g\right)}-\frac{\sum_{}^{}\left(p+g-2pg\right)}{{\left[\sum_{}^{}\left(p+g\right)\right]}^{2}}$$

where *P* denotes the predicted salient target map, while *p* denotes a pixel in this map. Equation (17) demonstrates that the gradient of the consistency enhancement loss is related to the pixel category, resulting in the same gradient for pixels of the same category and different gradients for pixels of different categories. This difference can increase the contrast between the foreground and the background, making the internal pixels of both foreground and background more uniform.

(2) Saliency Boundary Detection Loss (SBDL)

The imbalance between the number of boundary pixels and non-boundary pixels, caused by the high sparsity of boundary pixels, presents a challenge in the supervision of the salient boundary learning process. To address this issue, a balanced binary entropy loss is employed. This loss function ensures that the supervision is not biased towards any pixel type, hence solving the problem of pixel imbalance. The specific expression of the balanced binary entropy loss function is shown in Equation (18).

(18) $$L_{edge}=\sum_{p\subseteq P,g\subseteq G}^{}-\left[\left(1-\beta \right)\left(1-g\right)log\left(1-p\right)+\beta glogp\right]$$

where β represents the proportion of non-boundary pixels to all pixels.

## 4. Experiments and Analysis

Below is the pseudo-code for the proposed SOD method based on boundary enhancement and multi-scale information extraction:

# Function for Salient Object Detection

def salient_object_detection (image):

# Step 1: Preprocess the input image (e.g., normalization, resizing)

preprocessed_image = preprocess (image)

# Step 2: Extract features using a pre-trained backbone model (e.g., VGG16 or ResNet50)

features = extract_features (preprocessed_image)

# Step 3: Perform boundary enhancement

enhanced_features = boundary_enhancement (features)

# Step 4: Multi-level feature aggregation

aggregated_features = multi_level_feature_aggregation (enhanced_features)

# Step 5: Multi-scale information extraction

extracted_info = multi_scale_information_extraction (aggregated_features)

# Step 6: Boundary extraction

boundary_info = boundary_extraction (extracted_info)

# Step 7: Fusion of boundary and salient target information

fused_info = fusion (boundary_info, extracted_info)

# Step 8: Saliency prediction

saliency_map = predict_saliency (fused_info)

return saliency_map

Provided below are the detailed descriptions:

(1) preprocess (image): This function preprocesses the input image to prepare it for feature extraction. Common preprocessing steps include normalization, resizing, and data formatting to match the input requirements of the pre-trained backbone model.

(2) extract_features (preprocessed_image): This function extracts high-level features from the preprocessed image using a pre-trained backbone model such as VGG16 or ResNet50. The backbone model captures rich image representations that serve as the basis for subsequent processing.

(3) boundary_enhancement (features): In this step, the features obtained from the backbone model are further enhanced by incorporating boundary information. The method aims to improve the completeness and distinctiveness of salient object boundaries, making use of edge-aware techniques to highlight sharp transitions and edges in the image.

(4) multi_level_feature_aggregation (enhanced_features): This function aggregates features at multiple levels to handle large-scale variations of salient objects. By combining adjacent features and leveraging complementary information, the expressive ability of fixed-resolution features is enhanced.

(5) multi_scale_information_extraction (aggregated_features): The multi-scale information extraction module captures local contextual information at different scales for the aggregated features. This helps measure the composition of the feature map after back-fusion, leading to more accurate saliency predictions.

(6) boundary_extraction (extracted_info): The boundary extraction module processes the extracted information to identify and extract boundary information of salient regions. This is crucial for refining the saliency prediction results, especially in regions with low confidence.

(7) fusion (boundary_info, extracted_info): The fusion process combines the boundary information with the extracted salient target information. This fusion step refines the saliency prediction results by incorporating boundary cues, leading to improved accuracy.

(8) predict_saliency (fused_info): Finally, the fused information is used for saliency prediction, resulting in a saliency map highlighting the most visually significant regions in the input image.

### 4.1. Datasets and Evaluation Metrics

This paper presents experiments conducted on four publicly available datasets. The first dataset used in this study is DUTS [14], which is the largest saliency dataset available. It is comprised of 10,553 training images and 5019 test images and is known for its complex saliency target scenes. Many salient object detection methods in recent years have employed the DUTS training set to train their models. The second dataset is DUT-OMRON [15], which consists of 5168 images containing singular or plural salient objects, diverse image content, and complex background. Apart from pixel-level annotations for salient object detection tasks, the dataset includes bounding box annotations and gaze point data, which can be used for object localization and gaze point prediction. The third dataset used in the experiments is HKU-IS [16], comprised of 4447 images characterized by multiple disconnected salient objects and inconspicuous contrast between objects and background. Lastly, the ECSSD [17] dataset used in this study is an extension of the CSSD dataset. It includes 1000 images with rich semantics but complex structures sourced from the BSD dataset, PASCAL VOC dataset, and image data on the Internet.

The experiments conducted in this paper employ five widely used evaluation metrics for salient object detection tasks. The precision–recall (PR) curve, which was often used in early studies, measures precision and recall by calculating the binarized predicted output and the true value map of the image. The formula for the calculation process is shown in Equation (19):

(19) $$Precision=\frac{TP}{TP+FP},\;\;Recall=\frac{TP}{TP+FN}$$

where TP represents the number of correctly predicted salient pixels, FP represents the number of background pixels that are incorrectly predicted as significant points, and FN represents the number of incorrectly predicted background pixels. The threshold selection range for the indicator is between 0 and 255. A set of *P*, *R* values is obtained by outputting the binarization according to each threshold pair and then using the binary image and the corresponding true image to calculate the *P*, *R* values. The PR curve is plotted with Recall as the abscissa and Precision as the ordinate based on the average *P* and *R* values of all images under each threshold value.

F-measure: This metric is a weighted harmonic mean of precision and recall, and its calculation Equation (20) is expressed as follows:

(20) $$F_{\beta }=\frac{(1+\beta^{2})\times Precision\times Recall}{\beta^{2}\times Precision+Recall}$$

where $\beta^{2}$ is the weight parameter of precision and recall, which is usually set to 0.3 in practical applications to emphasize accuracy. We calculate the maximum $F_{\beta }$, defined as $F_{max}$, by taking the *P* value and *R* value from the PR curve. The larger the $F_{max}$, the better the performance of the model.

Mean Absolute Error (MAE): MAE takes into account all pixels and reflects the similarity between the predicted image and the ground truth image. The calculation Equation (21) of MAE is expressed as follows:

(21) $$MAE=\frac{1}{H\times W}\sum_{r=1}^{H}\sum_{c=1}^{W}\left|S\left(r,r\right)-G\left(r,c\right)\right|$$

where *H* and *W* represent the height and width of the image, respectively, while S(r,c) and G(r,c) respectively represent the predicted and ground truth values of the pixel located at (r,c). The smaller the MAE score, the better the performance.

E-measure [18]: It combines local pixel values with the image-level mean to capture both image-level statistical information and local pixel-matching information. Its calculation Equation (22) is expressed as follows:

(22) $$Q_{S}=\frac{1}{W\times H}\sum_{i=1}^{W}\sum_{j=1}^{H}\phi_{S}\left(i,j\right)$$

where $\phi_{S}\left(\cdot ,\cdot \right)$ represents an enhanced alignment matrix, which can be used to combine local and image-level information.

S-measure: This combines structural similarity at the region-aware level and object-aware level, supporting the acquisition of foreground structure in salient regions. It overcomes the shortcomings of evaluation criteria based on the pixel level that cannot obtain structural information. The calculation Equation (23) is as follows:

(23) $$S=\alpha \times S_{o}+\left(1-\alpha \right)\times S_{r}$$

where $S_{o}$ refers to the structural similarity of object perception, $S_{r}$ refers to the structural similarity of region perception and α is set to 0.5.

### 4.2. Experimental Environment

Experiments were implemented based on the PyTorch framework, and all experiments were completed on an NVIDIA GeForce RTX 3090 graphics card with 24GB of memory. The experiments in this section trained models on the DUTS training set and used random horizontal flips to augment the dataset during the training phase to avoid overfitting. The number of training cycles was 24 and the batch size was 1. The parameters were updated once every 10 samples’ gradients accumulated. The input image size during the training and testing phases was the original size. This section used a pre-trained VGG-16 or ResNet-50 model to initialize the parameters of the main feature extraction network, and the remaining parameters were initialized with a normal distribution. When the main network was VGG-16, the network used the Adam optimizer for gradient descent, and the related learning rate was $1\times 10^{-4}$, the weight decay was $5\times 10^{-4}$, and the learning rate was one-tenth of the original when the cycle number was 8 or 16. When the main network was ResNet-50, the learning rate changed to $5\times 10^{-5}$. The network was trained until the loss converged, and the entire training duration was 15 h. The ablation experiments were conducted on the DUTS dataset with VGG-16 as the backbone.

### 4.3. Ablation Experiment

This section proposes a boundary-enhanced salient object detection method for salient object detection tasks. To address the problem of significant scale variations of the target, this section proposes a multi-level feature aggregation module on the extracted features of the encoder to aggregate features of different levels and enhance the representation ability of features. Additionally, this section inserts a multi-scale information extraction module into each level of the decoder’s fusion to further extract context information of different scales. To address the issue of blurred boundary pixels of salient objects, this section proposes a boundary extraction module to extract salient boundary information on the boundary branch and then further supplement the salient object information. Furthermore, this section uses a set of mixed loss functions, which includes BCE for individual pixels and CEL for foreground areas, to supervise the model from different perspectives during training. To validate the effectiveness of the innovative points in this section’s method, detailed ablation experiments and analysis were conducted. The ablation experiments included structural ablation experiments for different modules and loss function ablation experiments. This section used the VGG-16 backbone FPN network structure as the baseline and binary cross-entropy as the loss function. The dataset used for the ablation experiments was the DUTS dataset, and the metrics included MAE, maximum F-measure, E-measure ($E_{m}$), and S-measure ($S_{m}$). The results of the two sets of experiments are shown in Table 1 and Table 2, respectively.

Structural ablation experiments: In order to evaluate the effectiveness of the proposed modules, a quantitative comparison of the results obtained using different modules was performed. The structural ablation experiment adds a separate multi-level feature aggregation module, multi-scale information extraction module, and boundary extraction module to the Baseline, and then further adds their two-by-two combinations and three combinations for experimentation. The results of the structural ablation experiment are shown in Table 1. It can be seen that all modules have significant performance improvements, with the single multi-scale information extraction module having the best performance. Compared to the Baseline, the MAE indicator is improved by 20.6%, the $F_{max}$ indicator is improved by 5.7%, the $E_{m}$ indicator is improved by 4.2%, and the $S_{m}$ indicator is improved by 2.3%. The combination of two modules has a further improvement compared to the single module, and the performance is best when all three modules are used together, with a 30.2%, 7.1%, 5.3%, and 3.5% improvement in MAE, $F_{max}$, $E_{m}$, and $S_{m}$, respectively.

**Figure 6:**
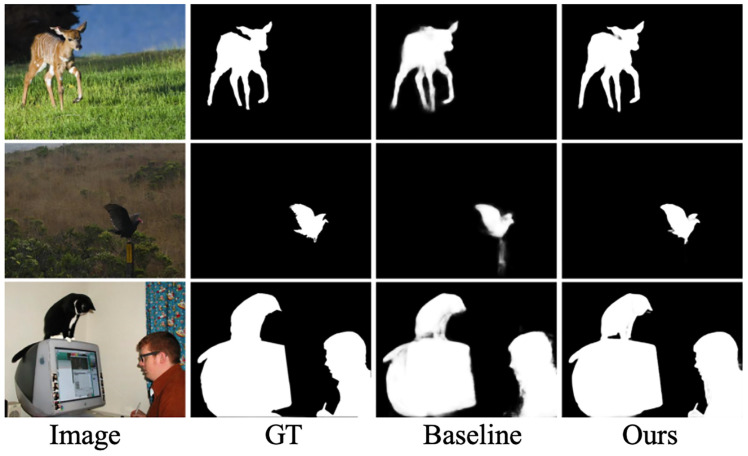
A visualization of the ablation experiment. From left to right are the original image, the truth image, the baseline result, and the result of this study.

Loss function ablation experiment: In the premise that all modules are equipped, single loss function and the combination of two loss functions were used respectively in the loss function ablation experiment. As can be seen from Table 2, CEL loss has a very large improvement on the model, and the effect is highest when two losses are mixed. The MAE, $F_{max}$, $E_{m}$, and $S_{m}$ indicators are improved by 6.8%, 0.8%, 1.4%, and 0.2%, respectively, achieving the best results in this section. In order to show the effect more intuitively, this section further visualizes the detection results of Baseline and this section’s method, as shown in Figure 6. It can be seen that the method of this section is significantly better than the Baseline method and not only can detect targets of different scales, but also has clear boundaries and uniformly consistent internal regions.

### 4.4. Comparative Experiments with Existing Methods

In order to demonstrate the superior performance and fairness of the proposed method, this section conducted comparisons on four common salient object datasets, including DUTS-TE, DUT-OMRON, HKU-IS, and ECSSD, as well as 11 mainstream methods based on VGG-16 or ResNet-50 in the past three years, including AFNet [19], PAGE [20], MLMSNet [21], CPD [22], GateNet [23], ITSD [24], AMPNet [25], EGNet [6], BANet [26], BASNet [9], and DNA [27]. The evaluation metrics used in all comparison experiments included $E_{m}$, $S_{m}$, MAE, and the maximum F-measure. It is worth noting that all the results of the comparative experiments are from the original literature or calculated from the saliency detection maps provided by the authors. The comparison results based on VGG-16 and ResNet-50 backbones are shown in Table 3, Table 4, Table 5 and Table 6, respectively.

From Table 3 and Table 4, it can be seen that the method in this section based on VGG-16 as the backbone has achieved advanced results on the four evaluation indicators of the four datasets. When using MAE as a reference, the method in this section is better than all the comparison methods, and the average improvement over the methods with slightly worse performance in each dataset is 3.6%. When using $F_{max}$ as a reference, the method in this section is better than other methods in three datasets and ranks second in the DUTS-ORMON dataset, with an average improvement of 0.2% relative to the methods with slightly worse performance in the three datasets with the best performance. When using Em as a reference, the method in this article leads all comparison methods on DUTS-TE and HKU-IS, with an average improvement of 0.3%. It ranks second in the other two datasets, but the difference from the best method is only 0.2% on average. When using Sm as a reference, the method in this article leads all comparison methods on the HKU-IS dataset, with an improvement of 0.2% compared to the best comparison method. It ranks second on the DUTS-TE and DUTS-OMRON datasets, but the average difference from the best method is only 0.5%. Analysis of the results shows that the DUTS-ORMON and ECSSD datasets contain many visually significant objects with complex structures and diverse contents, while the method in this section focuses more on solving the problems of scale variation and boundary blur, and imposes constraints at the pixel and image levels. Therefore, it does not achieve the best performance on the $E_{m}$ index that focuses on local pixel information and the $S_{m}$ index that focuses on local structural similarity. In addition, when VGG-16 is used as the backbone, the learning ability of the network model is limited and cannot fully utilize the performance of the module, resulting in poor performance on some data, but it is still very competitive.

**Figure 7:**
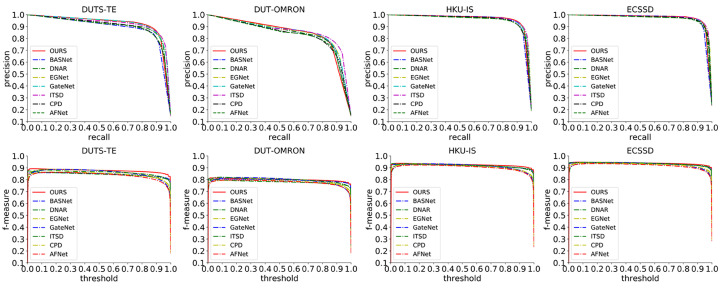
PR curves and F-measure curves.

It can be seen from Table 5 and Table 6 that the proposed method in this study based on ResNet-50 as the backbone achieves greatly advanced results on the four evaluation metrics of the four datasets. When using the MAE metric as a reference, the proposed method outperforms all comparison methods, with an average improvement of 6.2% compared to the second-best method in each dataset. Similarly, when using the Em metric as a reference, the proposed method also outperforms all comparison methods, with an average improvement of 0.7% compared to the second-best method in each dataset. When using the $F_{max}$ metric as a reference, the proposed method outperforms existing methods on three datasets and achieves an average improvement of 0.3% compared to the second-best method in the best three datasets. When using the Sm metric as a reference, the proposed method only outperforms all comparison methods on the DUTS-TE and HKU-IS datasets, with an average improvement of 0.2%. The analysis results show that using a more complex ResNet-50 as the backbone can further improve the performance of the module compared to using VGG-16 as the backbone, indicating that more complex backbone networks can further enhance the performance of the module. The experiment did not achieve the best results among the comparison methods in the $F_{max}$ metric on DUTS-OMRON, and the performance on the Sm metric on DUTS-OMRON and ECSSD was average. This is mainly because the proposed method focuses more on solving the problems of scale variation and boundary blur, and there is still room for further improvement in handling complex structured image data. In addition, this section also shows the PR curve and F-measure curve comparisons between the proposed method and other mainstream methods in Figure 7. It can be seen that the proposed method achieves advanced results on the DUTS-TE, HKU-IS, and ECSSD datasets, and it is also very competitive on the DUT-OMRON dataset.

**Figure 8:**
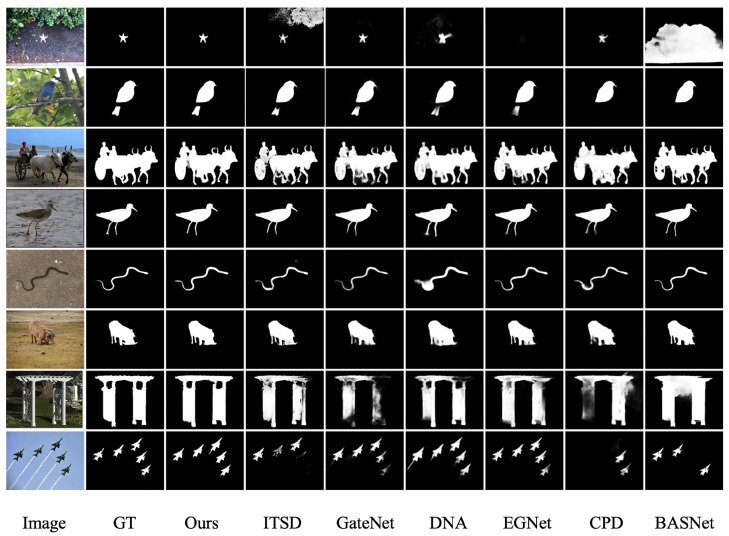
The visualization comparison chart of the comparative experiment. The left two columns display the original image and the ground truth image, while the remaining columns show the results of the proposed method and other methods.

Table 3, Table 4, Table 5 and Table 6 and Figure 7 demonstrate the superior performance of our method in terms of quantitative metrics such as F-measure and MAE. As shown in Figure 8, in order to more intuitively demonstrate the significant object detection performance of our method, this section presents visualizations of the detection results of our method and other comparison methods in some scenarios.

Figure 8 displays various scenes, including large objects, small objects, multiple objects, linear objects, partially occluded objects, and complex scenes. It can be seen from the figure that our method not only highlights the complete salient region, but also detects clear boundaries, while ensuring that the predicted salient region has uniformly consistent pixels inside.

Regarding the analysis of Laguerre–Gaussian beams in both monochromatic [28] and polychromatic cases [29,30], as well as beams passing through turbulent atmosphere [31,32], it is believed that the SOD method has the potential to be applied in these areas. SOD techniques can play a crucial role in identifying and analyzing important regions or features in complex optical wavefronts, including Laguerre–Gaussian beams. The ability to accurately detect and analyze salient regions in these optical structures can provide valuable insights into their characteristics and behavior.

While the proposed method has demonstrated promising performance in diverse settings, it is acknowledged that there is a need for tailored adaptations and further investigations to address the specific challenges presented by Laguerre–Gaussian beams and beams affected by turbulent atmosphere. These scenarios may require modifications in the feature extraction process, boundary enhancement, and multi-scale information extraction to account for unique optical properties and disturbances. Exploring such adaptations and conducting thorough experimental validations in these specific research areas are avenues for future research.

The limitations and challenges of the proposed approach lie in the following points. (1) Boundary Extraction Accuracy: The performance of the proposed method heavily relies on the accuracy of the boundary extraction module. If the boundary information is not accurately captured, it may lead to suboptimal saliency maps and affect the overall performance of the method. (2) Sensitivity to Noise: The method’s performance might be sensitive to noise in the input data, which could result in false positives or false negatives in the saliency maps. Addressing noise robustness is essential for achieving consistent and reliable results. (3) Computational Complexity: The proposed approach involves multiple stages, including edge extraction, multi-level feature aggregation, and multi-scale information extraction. This might lead to increased computational complexity, making real-time applications challenging on resource-constrained devices.

Future research directions for the SOD method based on boundary enhancement lie in the following points. (1) End-to-End Learning: Exploring end-to-end learning approaches could streamline the salient object detection pipeline, reducing computational complexity and potentially improving performance by jointly optimizing all modules. (2) Attention Mechanisms: Investigating the integration of attention mechanisms into the proposed approach could enhance the model’s focus on salient regions, leading to improved saliency map quality. (3) Saliency Propagation: Researching techniques for propagating saliency information through hierarchical architectures could enable more precise localization of salient objects at different scales. (4) Saliency in Videos and 3D Data: Extending the proposed method to video data and 3D point cloud data would open up new applications, such as video object segmentation and 3D scene understanding.

## 5. Conclusions

This paper proposes a salient object detection method based on boundary enhancement to solve the problem of large object scale variation and blurred border area pixels in the salient object detection task. This method has four main innovations: First, this method proposes a multi-level feature aggregation module for horizontal connection, using feature information of different resolutions to complement each other, and further enhancing the expressive ability of single-resolution features. Second, this method proposes a multi-scale information extraction module, which is inserted into the fusion process of each stage of the decoder to extract information of different scales from fixed-resolution features to better solve the problem of large changes in the target scale. Third, this method explicitly models the saliency boundary, uses the boundary extraction module to extract the boundary information and further supplements the salient target feature information, which solves the problem of unclear boundary pixels to a certain extent. Fourth, the method uses a hybrid loss function to supervise model training at different levels to highlight salient regions more uniformly. The experimental results show that the method can achieve competitive performance on four common saliency datasets and also outperforms current mainstream methods on multiple indicators.

## Figures and Tables

**Table 1:** Structural ablation experiment results.

Multi-Level Aggregation	Multi-Scale	Boundary Extraction	$F_{\max}$ ↑	$E_{m}$ ↑	$S_{m}$ ↑	MAE ↓
			0.812	0.852	0.839	0.063
*√*			0.85	0.883	0.856	0.054
	*√*		0.858	0.888	0.858	0.050
		*√*	0.847	0.874	0.845	0.053
*√*	*√*		0.865	0.893	0.867	0.045
*√*		*√*	0.855	0.887	0.858	0.047
	*√*	*√*	0.870	0.891	0.861	0.045
*√*	*√*	*√*	**0.870**	**0.897**	**0.868**	**0.044**

**Table 2:** Loss function ablation experiment results.

BCE	CEL	$F_{\max}$ ↑	$E_{m}$ ↑	$S_{m}$ ↑	MAE ↓
*√*		0.870	0.897	0.868	0.044
	*√*	0.872	0.898	0.868	0.042
*√*	*√*	**0.877**	**0.910**	**0.870**	**0.041**

**Table 3:** Comparative experiments on DUTS-TE and DUTS-OMRON, using VGG-16 as the backbone.

Model	DUTS-TE				DUTS-OMRON			
	$F_{\max}$ ↑	$E_{m}$ ↑	$S_{m}$ ↑	MAE ↓	$F_{\max}$ ↑	$E_{m}$ ↑	$S_{m}$ ↑	MAE ↓
Ours	**0.877**	**0.910**	0.870	**0.041**	0.801	0.865	0.826	**0.055**
AFNet [19]	0.863	0.895	0.867	0.046	0.797	0.860	0.824	0.057
PAGE [20]	0.838	0.886	0.854	0.052	0.792	0.860	0.825	0.062
MLMSNet [21]	0.852	0.863	0.862	0.049	0.774	0.839	0.809	0.064
CPD [22]	0.864	0.908	0.867	0.043	0.794	**0.868**	0.818	0.057
GateNet [23]	0.870	-	0.869	0.045	0.794	-	0.820	0.061
ITSD [24]	0.876	-	**0.877**	0.042	**0.802**	-	**0.828**	0.063
AMPNet [25]	0.859	-	0.858	0.045	0.791	-	0.814	0.058

**Table 4:** Comparative experiments on HKU-IS and ECSSD, using VGG-16 as the backbone.

Model	HKU-IS				ECSSD			
	$F_{\max}$ ↑	$E_{m}$ ↑	$S_{m}$ ↑	MAE ↓	$F_{\max}$ ↑	$E_{m}$ ↑	$S_{m}$ ↑	MAE ↓
Ours	**0.931**	**0.952**	**0.909**	**0.031**	**0.941**	0.942	0.913	**0.039**
AFNet [19]	0.925	0.949	0.906	0.031	0.941	0.942	0.913	0.039
PAGE [20]	0.920	0.948	0.904	0.036	0.931	**0.943**	0.912	0.042
MLMSNet [21]	0.920	0.938	0.907	0.039	0.928	0.916	0.911	0.045
CPD [22]	0.924	0.952	0.904	0.033	0.936	**0.943**	0.910	0.040
GateNet [23]	0.928	-	0.909	0.035	0.941	-	**0.917**	0.041
ITSD [24]	0.927	-	0.906	0.035	0.939	-	0.914	0.040
AMPNet [25]	0.920	-	0.896	0.034	0.937	-	0.909	0.040

**Table 5:** Comparative experiments on DUTS -TE and DUTS-OMRON, using ResNet-50 as the backbone.

Model	DUTS-TE				DUTS-OMRON			
	$F_{\max}$ ↑	$E_{m}$ ↑	$S_{m}$ ↑	MAE ↓	$F_{\max}$ ↑	$E_{m}$ ↑	$S_{m}$ ↑	MAE ↓
Ours	**0.893**	**0.928**	**0.887**	**0.034**	0.815	**0.876**	0.837	**0.050**
EGNet [6]	0.889	0.907	0.886	0.039	0.815	0.874	**0.841**	0.053
CPD [22]	0.865	0.904	0.869	0.043	0.797	0.873	0.825	0.056
BANet [26]	0.872	0.907	0.879	0.040	0.803	0.865	0.832	0.059
BASNet [9]	0.859	0.884	0.866	0.048	0.805	0.869	0.836	0.056
GateNet [23]	0.888	0.903	0.884	0.040	0.818	0.868	0.837	0.055
ITSD [24]	0.883	0.898	0.883	0.041	**0.821**	0.867	0.839	0.061
DNA [27]	0.874	0.907	0.870	0.040	0.807	0.863	0.825	0.056
AMPNet [25]	0.861	-	0.860	0.041	0.782	-	0.809	0.055

**Table 6:** Comparative experiments on HKU-IS and ECSSD, using ResNet-50 as the backbone.

Model	HKU-IS				ECSSD			
	$F_{\max}$ ↑	$E_{m}$ ↑	$S_{m}$ ↑	MAE ↓	$F_{\max}$ ↑	$E_{m}$ ↑	$S_{m}$ ↑	MAE ↓
Ours	**0.937**	**0.959**	**0.917**	**0.027**	**0.948**	**0.953**	0.922	**0.033**
EGNet [6]	0.935	0.956	0.917	0.031	0.947	0.947	**0.925**	0.037
CPD [22]	0.925	0.952	0.906	0.034	0.939	0.949	0.918	0.037
BANet [26]	0.930	0.955	0.913	0.032	0.945	0.953	0.924	0.035
BASNet [9]	0.930	0.947	0.908	0.033	0.942	0.921	0.916	0.037
GateNet [23]	0.933	0.953	0.915	0.033	0.945	0.943	0.920	0.040
ITSD [24]	0.934	0.953	0.917	0.031	0.947	0.932	0.925	0.034
DNA [27]	0.934	0.955	0.913	0.028	0.944	0.951	0.918	0.035
AMPNet [25]	0.918	-	0.895	0.033	0.937	-	0.910	0.038

## Data Availability

All the datasets used in this manuscript are publicly available datasets, already in the public domain.
